# Boosting of Antioxidants and Alkaloids in *Catharanthus roseus* Suspension Cultures Using Silver Nanoparticles with Expression of *CrMPK3* and *STR* Genes

**DOI:** 10.3390/plants10102202

**Published:** 2021-10-17

**Authors:** Ahmed Fouad, Adel E. Hegazy, Ehab Azab, Ebtihal Khojah, Tarek Kapiel

**Affiliations:** 1 Botany and Microbiology Department, Faculty of Science, Cairo University, Cairo 12613, Egypt; ahmedsfouad@yahoo.com; 2 Department of Plant Biotechnology, Genetic Engineering and Biotechnology Research Institute, University of Sadat City, Sadat City 32897, Egypt; amradel807080@googlemail.com; 3 Department of Food Science and Nutrition, College of Sciences, Taif University, P.O. Box 11099, Taif 21944, Saudi Arabia; e.azab@tu.edu.sa (E.A.); eykhojah@tu.edu.sa (E.K.)

**Keywords:** silver nanoparticles, *Catharanthus roseus*, H_2_O_2_, malondialdehyde, APX, SOD, *CrMPK3*, *STR*, alkaloids

## Abstract

Global agricultural systems are under unprecedented pressures due to climate change. Advanced nano-engineering can help increase crop yields while ensuring sustainability. Nanotechnology improves agricultural productivity by boosting input efficiency and reducing waste. Alkaloids as one of the numerous secondary metabolites that serve variety of cellular functions essential for physiological processes. This study tests the competence of silver nanoparticles (AgNPs) in boosting alkaloids accumulation in *Catharanthus roseus* suspension cultures in relation to the expression of *C. roseus* Mitogen Activated Protein Kinase 3 (*CrMPK3*) and Strictosidine Synthase (*STR*) genes. Five concentrations (5, 10, 15, 20 and 25 mg·L^−1^) of AgNPs were utilized in addition to deionized water as control. Results reflected binary positive correlations among AgNPs concentration, oxidative stress indicated with increase in hydrogen peroxide and malondialdehyde contents, activities of ascorbate peroxidase and superoxide dismutase, expression of the regulatory gene *CrMPK3* and the alkaloid biosynthetic gene *STR* as well as alkaloids accumulation. These correlations add to the growing evidence that AgNPs can trigger the accumulation of alkaloids in plant cells through a signaling pathway that involves hydrogen peroxide and MAPKs, leading to up-regulation of the biosynthetic genes, including *STR* gene.

## 1. Introduction

*Catharanthus roseus*, belonging to Apocynaceae is an essential medicinal plant synthesizing about 130 precious indole alkaloids [1], many of which are important for valuable drugs [2]. The common precursor for monoterpenoid indole alkaloids (strictosidine) is produced in the shikimate pathway step catalyzed by strictosidine synthases (*STR*) [3]. However, the low productivity and high cost of alkaloids chemical synthesis put *C. roseus* as an alternative source for industrial extraction of such invaluable secondary metabolites [4,5].

Plant secondary metabolites, including alkaloids, are important for the plant to interact with its environment for adaptation and defense against different types of stress [6,7]. Heavy metals, at phytotoxic concentrations, bring about oxidative stress accompanied with motivation in reactive oxygen species (ROS) generation [8]. Besides provoking ascorbate-glutathione cycle enzymes and other antioxidant enzymes [e.g., catalase (CAT), peroxidases (POD), and superoxide dismutase (SOD)]. Metabolic activities are directed towards pathways consuming reduction equivalents. Consequently, the biosynthetic pathways of reduced compounds, including alkaloids, are promoted [9,10]. The utilization of heavy metals to enhance alkaloids biosynthesis in *C. roseus* was recorded using Mn^2+^, Pb^2+^, and Ni^2+^ [11], Hg^2+^ [12], Cu^2+^ [3], Cd^2+^ [13], Ag^+^ [14], Co^2+^ [15] and Cr^2+^ [16].

Mitogen-activated protein kinases (MAPKs) are a major group of protein kinases playing a crucial role in coupling the perception of stressful stimuli with alterations in gene expression [17]. MAPK cascade is fundamental for alkaloids biosynthesis while experiencing stress conditions [18]. Up-regulation of several genes related to alkaloids biosynthetic pathway coupled with stimulation of *CrMPK3* expression was recorded in *C. roseus* following copper treatments [3] and silver exposure [14].

Although stress promotes alkaloids production, it is usually associated with a decrease in growth of the producing plant that may neglect an increase in the total amount of alkaloids synthesized per plant [15]. Thus, stress should be manipulated to produce the maximum enhancement in alkaloids production with minimum growth retardation. Applying heavy metals, such manipulation can be achieved through variations in the used metal concentration and stressful treatment duration. The unique characteristics of nanoparticles, compared with their bulk counterparts, may provide an additional tool to manipulate heavy metal-associated stress [19].

Silver nanoparticles (AgNPs) offer a unique biological activity and can function as new stimulators for plant growth [20]. Positive effects of AgNPs include germination stimulus, revitalization of growth, accumulation of biomass, improved induction and proliferation of shoots or higher pigment content [21,22,23]. Several authors documented the potential use of AgNPs- enhance accumulation of secondary metabolites in tissue cultures of hazel [24], *Cucumis anguria* [25], *Caralluma tuberculata* [26], and *Linum usitatissimum* [27]. Nevertheless, few studies [25,26] were devoted to monitoring the impact of AgNPs on alkaloids production in plant tissues and lacked elucidation for the genetic expression profile underlying the biosynthetic pathway.

Plant cell and tissue cultures are routinely utilized to study and enhance alkaloids production in *C. roseus* [28]. However, cell suspension culture is the start point for the establishment of bioreactors and commercial production of the precious metabolites [29].

The aim of this work is to study the positive potential effect of using AgNPs in the stimulation of alkaloids biosynthesis in *C. roseus* suspension cultures by triggering the expression of *CrMPK3* and *STR* genes.

## 2. Results

### 2.1. Growth Parameters

**Figure 1:**
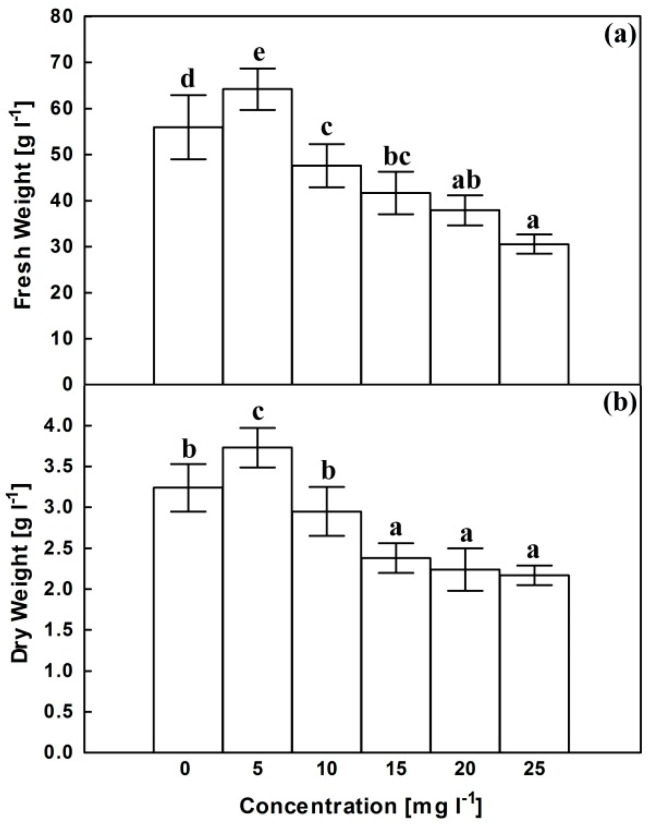
Effects of AgNPs at concentrations of 5, 10, 15, 20 and 25 mg L^−1^ on fresh weight (**a**) and dry weight (**b**) of *C. roseus* cell suspension cultures. Means ± standard deviations (SDs), n = 3, Values followed by the same small letter within each column are not significantly different at *p* ≤ 0.05.

Regarding the corresponding control, AgNPs have a growth-promoting effect at 5 mg.L^−1^ manifested in about 15% significant increase in both fresh and dry weights (Figure 1). Higher concentrations of AgNPs was associated with decrease in fresh weight reaching about 55% of the corresponding control at 25 mg L^−1^. Dry weight remained unaffected at 10 mg L^−1^ then decreased reaching about 73% of control at 15 mg L^−1^ that remained insignificantly changed with furthur increases in AgNPs concentration.

### 2.2. Hydrogen Peroxide (H_2_O_2_) Content and Lipid Peroxidation

**Figure 2:**
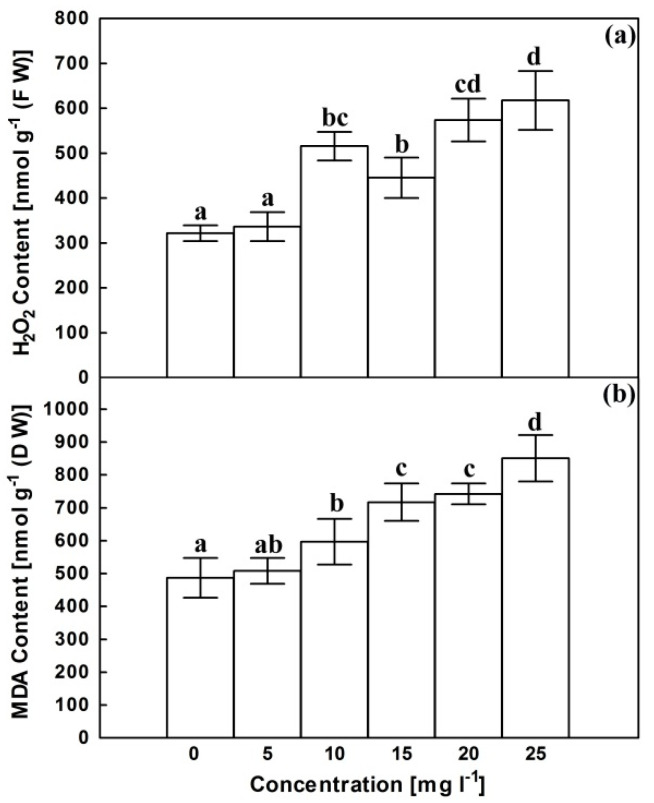
Effects of AgNPs at concentrations of 5, 10, 15, 20 and 25 mg L^−1^ on H_2_O_2_ content (**a**) and MDA content (**b**) of *C. roseus* cell suspension cultures. Means ± standard deviations (SDs), n = 3, Values followed by the same small letter within each column are not significantly different at *p* ≤ 0.05.

Compared with untreated cultures, AgNPs have no significant effect on H_2_O_2_ content at 5 mg L^−1^ (Figure 2a). However, the increase in AgNPs concentration was associated with a significant irregular increase in H_2_O_2_ content to be approximately doubled at 25 mg L^−1^. Lipid peroxidation, symbolized with malondialdehyde (MDA) content, exhibited a more or less similar trend following exposure to AgNPs (Figure 2b). It remained unaffected at 5 mg L^−1^ then increased at higher concentrations reaching about 1.75 fold control at 25 mg L^−1^.

### 2.3. Relative Expression of CrMPK3 and STR Genes

**Figure 3:**
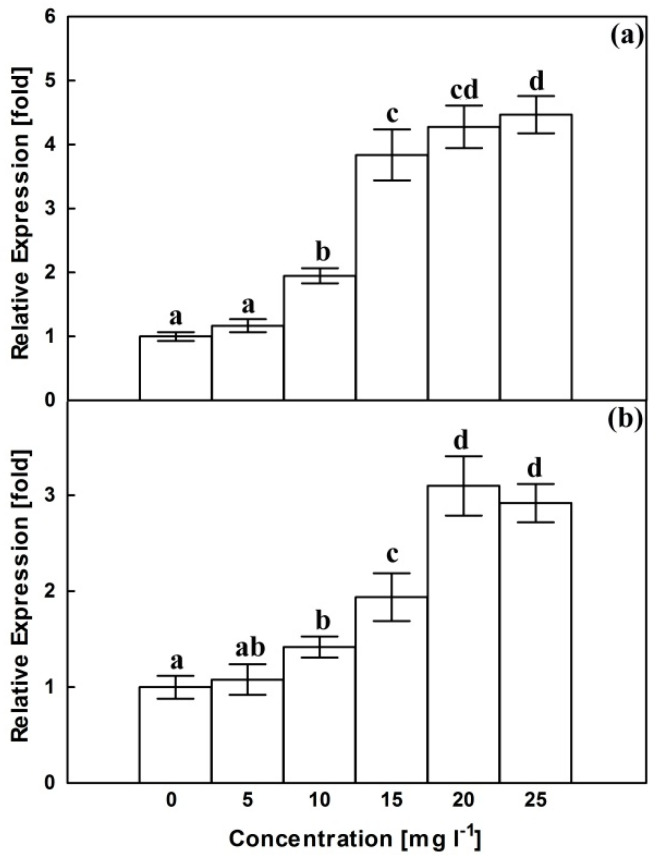
Effects of AgNPs at concentrations of 5, 10, 15, 20 and 25 mg L^−1^ on relative expression of CrMPK3 gene (**a**) and STR gene (**b**) of *C. roseus* cell suspension cultures. Means ± standard deviations (SDs), n = 3, Values followed by the same small letter within each column are not significantly different at *p* ≤ 0.05.

Compared with the corresponding control, AgNPs did not aggravate the expression of the CrMPK3 gene at 5 mg L^−1^ (Figure 3a). The transcript abundance increased dramatically at 10 and 15 mg L^−1^ then steeply at higher concentrations reaching about 4.5 folds of control at 25 mg L^−1^. Similar to CrMPK3 gene, STR gene expression remained insignificantly affected at 5 mg L^−1^, compared with control expression (Figure 3b). However, the higher AgNPs concentrations were associated with a significant increase in STR transcript to reach a maximum of 3.1 folds of control at 20 mg L^−1^.

### 2.4. Alkaloids Content

**Figure 4:**
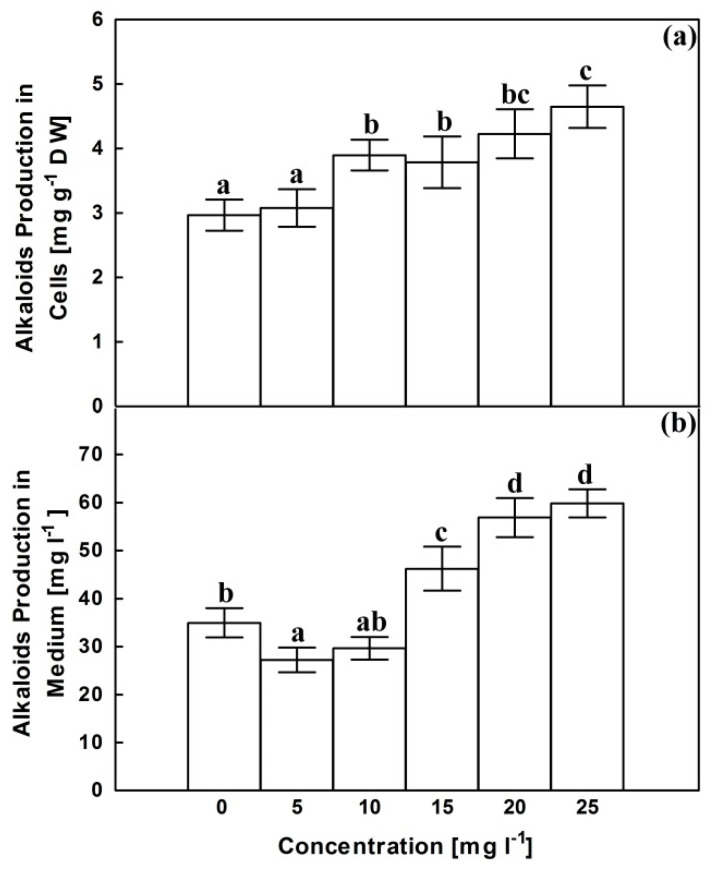
Effects of AgNPs at concentrations of 5, 10, 15, 20 and 25 mg L^−1^ on alkaloids content in dried cells (**a**) and growth medium (**b**) of *C. roseus* cell suspension cultures. Means ± standard deviations (SDs), n = 3, Values followed by the same small letter within each column are not significantly different at *p* ≤ 0.05.

Control cultures accumulate about 3 mg alkaloids in each gram of dried cells and about 35 mg othese secondary metabolites in each liter liquid growth medium after filtering the cells (Figure 4). Cellular accumulation of alkaloids remained insignificantly changed at 5 mg L^−1^; however, higher AgNPs concentrations were accompanied with significant enhancements in alkaloids accumulation in cells to reach 1.42 folds of the corresponding control at 25 mg L^−1^. Alkaloids accumulation in growth medium declined significantly at 5 mg L^−1^ and remained comparaple with corresponding control upon exposure to AgNPs at 10 mg L^−1^. Above 10 mg L^−1^, AgNPs significantly enhanced accumulation of alkaloids in growth medium reaching about 1.7 folds of the corresponding control at 25 mg L^−1^.

### 2.5. Antioxidant Enzymes

**Figure 5:**
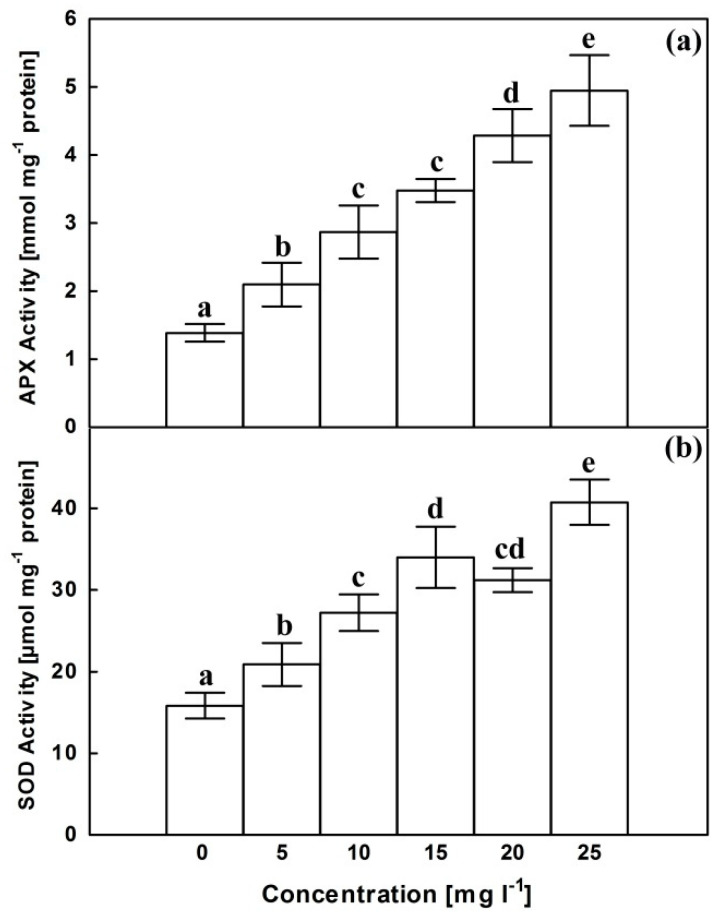
Effects of AgNPs at concentrations of 5, 10, 15, 20 and 25 mg L^−1^ on APX activity (**a**) and SOD activity (**b**) of *C. roseus* cell suspension cultures. Means ± standard deviations (SDs), n = 3, Values followed by the same small letter within each column are not significantly different at *p* ≤ 0.05.

APX activity significantly increased more or less gradually following exposure to the increasing AgNPs concentrations reaching about 3.5 folds of control at 25 mg L^−1^ (Figure 5a). Assuming a slightly different trend, SOD activity increased in response to AgNPs treatments reaching a maximum of about 2.5 folds of control at 25 mg L^−1^ (Figure 5b).

Correlation analysis (Table 1) reflected significant negative Pearson correlations between AgNPs concentration and growth parameters, while all other concerned parameters appeared to be positively correlated with the concentration of AgNPs. Similar correlations were demonstrated for each of H_2_O_2_ and MDA contents with other parameters. Positive correlations were documented between CrMPK3 expression and STR expression as well as alkaloids content, either in cells or secreted in growth medium.

## 3. Discussion

Results of the present investigation reflected a biphasic effect of AgNPs on the growth of *C. roseus* cells. The available literatures provide contradictory results for the impact of AgNPs on plant growth [20]. Such effect seems to vary with particle size, shape, and concentration and is complicated with the treated plant material. The growth stimulation observed at low concentration, as shown in Figure 1, can be explained in light of the study conducted by Castro-González et al. [30] on stevia (*Stevia rebaudiana* B.) in vitro seedlings. The authors attributed the growth-promoting effect of low concentrations of AgNPs to inhibition of ethylene biosynthesis and improvement of nutrient accumulation and antioxidant metabolism.

In the same context, Gupta et al. [31] related the growth promotion recorded for *Oryza sativa* seedlings following AgNPs treatments to the acompanied increase in antioxidant enzymes activity that reduces oxidative stress and hydrogen peroxide content and prevents lipid peroxidation that appeared obviously in our results at 5 mg L^−1^. The inhibitory effect of AgNPs on ethylene biosynthesis [32] combined with the role of ethylene in signaling alkaloids production [33] can be used to explain the decrease in alkaloids accumulation in growth medium recorded in the current study.

Starting from 10 mg L^−1^, AgNPs showed a growth retarding effect (Figure 1) that can be attributed to the accompanied negatively correlated oxidative stress as manifested by an increase in H_2_O_2_ content and the resultant increase in lipid peroxidation products. These symptoms of oxidative stress were observed following exposure to AgNPs in rice seedlings [34], wheat callus [35], *Pisum sativum* seedlings [36], lettuce plants [37], *Salvia officinalis* seedlings [38], and *Allium cepa* root tips [39].

Recently, AgNPs have been a major revealer of bioactive molecule production. Many research reports have proven that the NPs could alter the secondary metabolism in plant and culture systems [40]. Despite the need for more research to understand the mechanism, some evidence suggests that NPs lead to the production of reactive oxygen species (ROS) and other second messengers, altering transcriptional regulation of plant secondary metabolism [41].

Plants typically produce H_2_O_2_ as a common metabolite for various cellular processes required for growth and development [42]. Nevertheless, H_2_O_2_ level rises significantly following exposure to different types of abiotic stress as a consequence of exaggerated energy and/or damaging of membrane system of chloroplast and mitochondria [43].

To cope with oxidative stress and to hunt the ROS, plants activate several metabolic pathways, including MAPK pathway, which is suggested as one of the plant’s early responses to AgNPs treatment [41]. Results of the current study reflected a positive correlation between expression of *CrMPK3* gene (Figure 3) and each of AgNPs concentration and H_2_O_2_ generation. H_2_O_2_–mediated induction of MAPK cascade was recorded in several plant species, including *Brassica napus* [44], *Nicotiana tabacum* [45], soybean leaves [46] and maize leaves [47]. H_2_O_2_ may activate MAPK pathway through the inactivation of MAPK repressors [48].

Induction of MAPK cascade transcriptionally elicits alkaloids biosynthesis to reduce ROS accumulation through their inhibitory effect on NADPH-oxidase, the key enzyme for ROS production [49]. Thus, our results reflected positive correlations between each of AgNPs concentration, H_2_O_2_ content, and expression of *CrMPK3* gene on one side and expression of *STR* gene as well as alkaloids content on the other side. Supporting these correlations, Khataee et al. [16] recorded that the enhancement of alkaloids accumulation following exposure to chromium was associated with an increase in lipid peroxidation and upregulation of MAPK and *STR* genes. In addition, Huerta-Heredia et al. [50] recorded an increase in alkaloids content in *Uncaria tomentosa* root cultures following exposure to H_2_O_2_. Working on the same plant, Vera-Reyes et al. [51] recorded the same findings in addition to documentation of accompanied-up regulation of *STR* gene.

The synchronization between up-regulation of *CrMPK3* and *STR* genes and increase in alkaloids accumulation in *C. roseus* was recorded following exposure to different types of elicitors, including wounding, UV and methyl jasmonate [52], ethylene and copper [3], and silver nitrate combined with methyl jasmonate [14]. In a similar context, Fouad and Hafez [15] recorded a positive correlation between expression of *CrMPK3* gene and alkaloids content in *C. roseus* suspension cultures experiencing oxidative stress symptoms following exposure to cobalt ions and cobalt nanoparticles.

The potential use of AgNPs to enhance alkaloids biosynthesis was proven in hairy root cultures of *Datura metel* [53] and *Isatis constricta* in vitro plants [54]. The increase in alkaloids content in cells without an associated increase in growth medium recorded in our results at 10 mg L^−1^ may reflect intracellular sinks for the newly synthesized alkaloids to cope the oxidative stress before immobilization to growth medium or suggest the ability of cells to retain alkaloids within intracellular specialized structures avoiding cytotoxicity with high concentrations in the growth medium [55].

**Figure 6:**
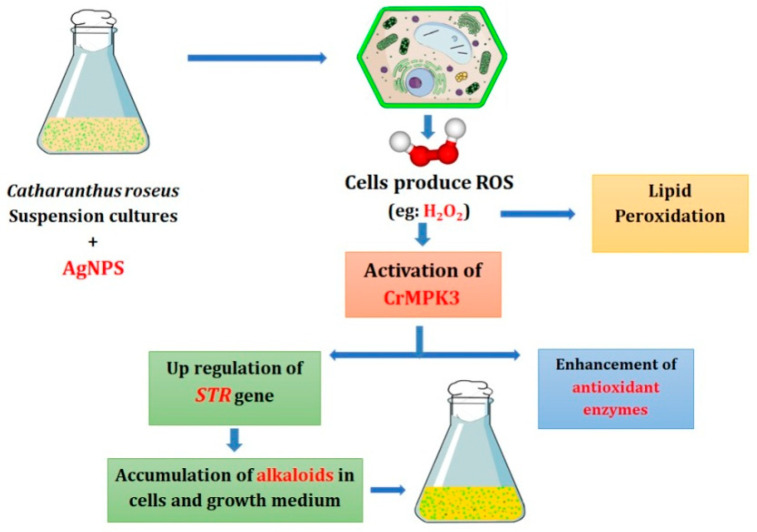
Summary for the action mechanism for AgNPs-mediated alkaloids biosynthesis in *Catharanthus roseus*.

Adding to antioxidant response against AgNPs-generated ROS, MAPKs stimulate expression and activities of antioxidant enzymes that are clearly demonstrated in positive correlations among AgNPs concentration, H_2_O_2_ content, expression of *CrMPK3* gene, and activities of APX and SOD in the present study. An increase in the activities of antioxidant enzymes following exposure to AgNPs was recorded by Nwaichi and Anosike [56] in *Vigna subterranea* plants, Barbasz et al. [35] in *Pisum sativum* seedlings, Ali et al. [26] in *Caralluma tuberculata* callus, and Jadczak et al. [57] in lavender in vitro plants. The involvement of MAPKs in the enhancement of antioxidant enzymes in response to oxidative stress is well documented in *C. roseus* suspension cultures [15], maize leaves [47], and wheat plants [58]. Summary for the action mechanism for AgNPs-mediated alkaloids biosynthesis in *Catharanthus roseus* in Figure 6 suggests induction of MAPKs genes that upregulate *STR* gene and enhances antioxidant enzemes.

## 4. Materials and Methods

### 4.1. Silver Nanoparticles Characterization and Dispersion

AgNPs were purchased from Nanotech, Cairo, Egypt, as roughly spherical 20–30 nm particles dispersed in deionized water (DW) in a concentration of 1mg/mL. Shape and size were confirmed with transmission electron microscopy (TEM) in our previous publication [59]. Experimental concentrations were prepared by diluting stock solution in DW, just before use, and sonication for 30 min at100 W and 30 kHz.

### 4.2. Plant Material and Explant Preparation

Healthy *C. roseus* seeds (generously afforded by Horticulture Research Institute, Agricultural Research Centre, Giza, Egypt) were surface sterilized sequentially in 70% (*v/v*) ethanol and 0.1% (*m/v*) HgCl_2_ for 1 and 10 min, respectively. Sterilized seeds were rinsed several times in sterile distilled water after that transferred into 350 mL jars (10– 15 seed per jar) containing germination medium. The medium was Murashige, and Skoog salts and vitamins [60] enrich with sucrose (30 gL^−1^) and solidified with agar (7 gL^−1^). The medium was autoclaved for 20 min at 121 °C after pH adjustment at 5.8. Cultures were incubated at 25 ± 2 °C and lighted at 30 μmol m^−2^ s^−1^ with 16/8 h photoperiod. After one week, seedlings were used as a source of hypocotyl explants.

### 4.3. Tissue Cultures and Silver Treatments

Cultures were established as described by Fouad and Hafez [61]. Calli were initiated on hypocotyl explants using callus induction medium of germination medium to which 0.5 mg.L^−1^ kinetin and 1.0 mgL^−1^ of each of 2,4- dichlorophenoxyacetic acid (2,4-D) and indole acetic acid (IAA) were added. Cultures were maintained in dark at 25 ± 2 °C for one month; then, the originated calli were subcultured on the same but fresh medium with time intervals of one month under the same growth conditions. Cell suspension cultures were initiated in 500 mL flasks, each containing 200 mL callus induction medium lacking agar. Each flask was aseptically inoculated with 10 g calli then placed on an orbital shaker at 130 rpm. Every 10 days, cultures were divided into 20 mL aliquots that transferred to 180 mL fresh medium for subculture. After one week of the fourth subculture, filter-sterilized aqueous AgNPs solution was aseptically introduced into cultures to reach a final concentration of 5, 10, 15, 20 and 25 mgL^−1^. In control cultures, AgNPs were replaced with DW. Cells were collected, by filtration, for different analyses 4 and 72 h after applying silver treatments. The 4 h treatment was dedicated for real-time quantitative PCR while 72 h treatment was devoted for other measurements. Fresh weights were quantified, then cells were crushed into a fine powder with the aid of liquid nitrogen and stored at −80 °C till used. Dry weights were determined after a drying step at 50 °C till unchangeable weight. Dried cells and liquid growth medium were utilized to quantify alkaloids accumulated in cells and excreted in medium, respectively.

### 4.4. H_2_O_2_

H_2_O_2_ content was quantified according to the method outlined by Loreto and Velikova [62]. 0.5 g cells were homogenized in 2.5 mL of freshly prepared trichloroacetic acid (0.1% (*w/v*)) in an ice bath. The homogenate was centrifuged for 20 min at 4 °C and 10,000 g. 0.5 mL of the clear supernatant was mixed with an equal volume of 10 mM potassium phosphate buffer (pH 7.0) and 1 mL of 1.0 M KI. The absorbance was recorded at 390 nm, and the amount of H_2_O_2_ (nmol g^−1^ fresh weight) was computed based on a standard curve constructed using different known concentrations of H_2_O_2_.

### 4.5. Lipid Peroxidation Assay

Lipid peroxidation was evaluated by quantification of malondialdehyde (MDA) formation employing the thio¬barbituric acid method Stewart and Bewley [63]. Liquid nitrogen-grinded cells belonging to 72 h treatment were blended with 100 mM Tris–HCl buffer, pH 7.4, containing 1.5% (*m/v*) polyvinylpyrrolidone (PVP). The homogenate was filtered, then the filtrate was centrifuged for 15 min at 15,000× *g*. 1 mL transparent supernatant was incubated at 90 °C with 4 mL of 20% (*m/v*) trichloroacetic acid containing thiobarbituric acid in a concentration of 0.5% (*m/v*). 30 min later, tubes were cooled and centrifuged for 15 min at 15,000× *g*. The MDA concentration was computed in the supernatant based on its extinction coefficient of 155 mM^−1^ cm^−1,^ and absorbance was read at 532 nm after subtraction of nonspecific absorbance at 600 nm.

### 4.6. Antioxidant Enzymes and Soluble Proteins

Stored frozen powder of 72 h treatment was mixed with extraction solution of 50 mM potassium phosphate buffer (pH 7.8), 0.2 mM EDTA and 1% PVP (*m/v*) [64]. The homogenate was centrifuged at 4 °C for 15 min at 20,000× *g* then the clear supernatant was used for assessment of ascorbate peroxidase (APX) and SOD activities as well as quantification of soluble proteins content.

Ascorbate peroxidase (APX) activity was assessed according to the protocol outlined by Nakano and Asada [65] using a reaction mixture of 50 mM phosphate buffer, pH 7.0, 0.5 mM ascorbate, 1.2 mM H_2_O_2,_ and 0.1 mM EDTA. The rate of decrease in oxidised ascorbate absorbance was recorded at 290 nm, and APX activity was computed using the extinction coefficient of 2.8 mM^−1^ cm^−1^ for ascorbate. SOD activity was evaluated according to the method described by Beauchami and Fridovich [66] through monitoring the inhibition of photochemical reduction of nitroblue tetrazolioum (NBT). The assay was conducted using a reaction mixture of 50 mM phosphate buffer (pH 7.8), 33 mM NBT, 0.66 mM Na-EDTA, 10 mM methionine, and 3.3 mM riboflavin at a light intensity of 300 μmol m^−2^ s^−1^. After 10 min at 25 °C, the change in OD was recorded at 560 nm. 50% inhibition in NBT reduction was defined as one SOD unit [67].

Soluble proteins were estimated at 750 nm as outlined by Lowry et al. [68] employing Folin-Ciocaltcau reagent and bovine serum albumin-based calibration curve.

### 4.7. Alkaloid Extraction and Determination

Alkaloid extraction and determination was carried out, as described by Lee et al. [69] for dry weights and broth of 72 h treatments. Fine powder of oven-dry cells was extracted three times in methanol. The three extracts were mixed then the solvent was vacuum-evaporated. In the separating funnel, the residue was shaken in acid water (pH 3) and petroleum ether. The aqueous phase was collected alkalinized to pH of 8.5 using 1 M NaOH, then total alkaloids were extracted with chloroform in a subsequent step. The collected liquid growth medium was acidified to pH 3 and shaked with petroleum ether; then extraction was proceeded as described for dried cells. Total alkaloids content was computed by comparing OD recorded at 280 nm with an ajmalicine (Sigma-Aldrich, St. Louis, MO, USA)-based standard curve.

### 4.8. Real-Time Quantitative PCR

Fresh cells collected from 4 h treatments were used for total RNA extraction with the aids of Direct-zol™ RNA MiniPrep (http://www.zymoresearch.com (accessed on 10 October 2020)). After a purification step, residual genomic DNA was eliminated using DNase (Fermentas, Waltham, MA, USA) then RNA purity and concentration were evaluated using Nanodrop spectrophotometer (ND−2000c, Thermo Fisher Scientific, Wilmington, DE, USA). One μg purified RNA was devoted for cDNA synthesis employing Sensi¬FAST™ cDNA synthesis kit (http://www.bioline.com (accessed on 10 October 2020)). *CrMPK3* and *STR* cDNAs were quantitatively amplified with the aid of a Mx3000P (Stratagene, CA, USA) qPCR system using specific primers (Table 2) [55]. The transcription level of actin was used as an endogenous control to which the transcription level of *CrMPK3* and *STR* genes were normalized using the 2^-ΔΔCt^ method [70]. The amplification protocol consisted of 40 amplification cycles (95 °C for 15 s and 60 °C for 60 s) preceded by 95 °C for 10 min. Expression estimated in cells collected from control cultures was employed as a quantification unit.

### 4.9. Statistical Analysis

Results of all treatments were exhibited as the mean of three replicates ± standard deviation (SD). Significant differences between different treatments was tested using the least significant difference (LSD) post-hoc test at level of significance set at *p* ≤ 0.05 using SPSS v. 14. Correlations between parameters were calculated using Minitab v. 10.0 software based on Pearson correlation at *p* values < 0.05.

## 5. Conclusions

Thus, we can conclude that exposure to AgNPs was associated with oxidative stress manifested in an increase in H_2_O_2_ content and lipid peroxidation products that provoked antioxidant defense through MAPK cascade represented by *CrMPK3*. The induced MAPK transcriptionally induces alkaloids biosynthetic genes, including *STR* gene enhancing alkaloids accumulation to prevent ROS formation. In addition, the induced MAPK provoked antioxidant enzymes to scavenge the generated ROS. The results suggest a mechanism for the potential role of AgNPs in the enhancement of alkaloids accumulation in plant cells through a signaling pathway that involves H_2_O_2_ and MAPKs, leading to up-regulation of the biosynthetic genes, including *STR* gene.

## Figures and Tables

**Table 1:** Pearson correlation matrix for AgNPs concentration and different evaluated parameters.

	AgNPs Conc.	Fresh Weight	Dry Weight	H_2_O_2_ Content	MDA Content	APX Activity	SOD Activity	*CrMPK3* Expression	*STR* Expression	Alkaloids in Cells
**Fresh Weight**	−0.874 *									
**Dry Weight**	−0.839 *	0.948 *								
**H_2_O_2_ Content**	0.883 *	−0.848 *	−0.790 *							
**MDA Content**	0.928 *	−0.848 *	−0.852 *	0.841 *						
**APX activity**	0.974 *	−0.813 *	−0.786 *	0.898 *	0.941 *					
**SOD activity**	0.933 *	−0.808 *	−0.767 *	0.753 *	0.890 *	0.907 *				
***CrMPK3*** **Expression**	0.950 *	−0.885 *	−0.883 *	0.809 *	0.904 *	0.928 *	0.883 *			
***STR*** **Expression**	0.926 *	−0.819 *	−0.854 *	0.828 *	0.869 *	0.908 *	0.776 *	0.904 *		
**Alkaloids in cells**	0.883 *	−0.802 *	−0.722 *	0.787 *	0.778 *	0.853 *	0.892 *	0.821 *	0.767 *	
**Alkaloids in medium**	0.860 *	−0.814 *	−0.844 *	0.685 *	0.835 *	0.834 *	0.763 *	0.902 *	0.927 *	0.751 *

* Correlation is significant at the 0.05 level.

**Table 2:** List of primers used for qRT-PCR (5′–3′).

Gene	Primers Sequence
Actin	5′-CTATGTTCCCAGGTATTGCAGATAGA-3′ 5′-GCTGCTTGGAGCCAAAGC-3′
*CrMPK3*	5′ACGAAATGAGGATGCAAAAAGATAC-3′ 5′-TGCTAACTGCTGACGAGGGAAT-3′
*STR*	5′-TGCTTCACTCCCATCATTTACAGT-3′ 5′-CTGCCATCATGGATTTAGATTCAG-3′

## Data Availability

Not applicable.
